# Virus Infection Variability by Single-Cell Profiling

**DOI:** 10.3390/v13081568

**Published:** 2021-08-09

**Authors:** Maarit Suomalainen, Urs F. Greber

**Affiliations:** Department of Molecular Life Sciences, University of Zurich, Winterthurerstrasse 190, CH-8057 Zurich, Switzerland

**Keywords:** single-cell infection, cell-to-cell variability, RNAseq, cell state, virus imaging, non-genetic variability, single transcript fluorescence in situ hybridization, click chemistry, virus entry, transcription, replication, assembly, egress, persistence and lysis

## Abstract

Cell-to-cell variability of infection has long been known, yet it has remained one of the least understood phenomena in infection research. It impacts on disease onset and development, yet only recently underlying mechanisms have been studied in clonal cell cultures by single-virion immunofluorescence microscopy and flow cytometry. In this review, we showcase how single-cell RNA sequencing (scRNA-seq), single-molecule RNA-fluorescence in situ hybridization (FISH), and copper(I)-catalyzed azide-alkyne cycloaddition (click) with alkynyl-tagged viral genomes dissect infection variability in human and mouse cells. We show how the combined use of scRNA-FISH and click-chemistry reveals highly variable onsets of adenoviral gene expression, and how single live cell plaques reveal lytic and nonlytic adenovirus transmissions. The review highlights how scRNA-seq profiling and scRNA-FISH of coxsackie, influenza, dengue, zika, and herpes simplex virus infections uncover transcriptional variability, and how the host interferon response tunes influenza and sendai virus infections. We introduce the concept of “cell state” in infection variability, and conclude with advances by single-cell simultaneous measurements of chromatin accessibility and mRNA counts at high-throughput. Such technology will further dissect the sequence of events in virus infection and pathology, and better characterize the genetic and genomic stability of viruses, cell autonomous innate immune responses, and mechanisms of tissue injury.

## 1. Introduction

**Figure 1:**
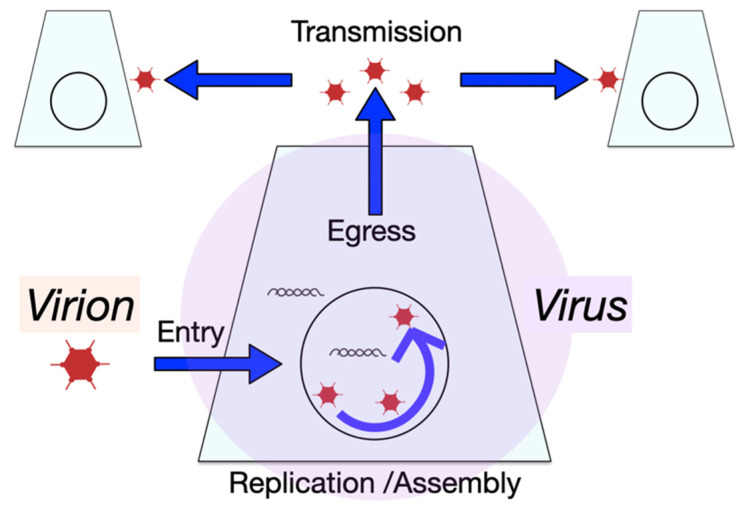
Schematic depiction of a virus-centric view into single-cell infection and dissemination of progeny to uninfected cells.

Virus infections are multi-step processes comprising entry, genome trafficking, activation of viral gene expression, replication of the genome, particle assembly, and release of progeny. Any virus infection depends on host factors, which are variably expressed in host cells. In fact, cell-to-cell variability in virus progeny production was reported as early as 1952 with a plaque assay of Western equine encephalomyelitis virus in chicken embryo cells by Dulbecco [1]. Infection variability comprises the multi-step virus entry process, the virion trafficking and dismantling, the initiation of gene expression or translation of the viral genome, the establishment of replication domains in the cytoplasm or the nucleus, and the subversion of egress pathways leading to progeny dissemination [2,3,4,5,6]. For a schematic depiction of a virus-centric view of infections, see Figure 1.

Classically, virus infection mechanisms have been studied by population-level assays, such as Western blot, RT-qPCR, bulk RNA sequencing of host transcriptomes, or bulk proteomics. Although the results from such assays have provided much of the foundation of molecular virology, these types of assays have considerable shortcomings. They provide a population-averaged view and hide important features, such as host gene expression differences between infected and uninfected cells, unproductively infected cells, or information on gene expression efficiency in specialized subsets of cells in a tissue.

**Figure 2:**
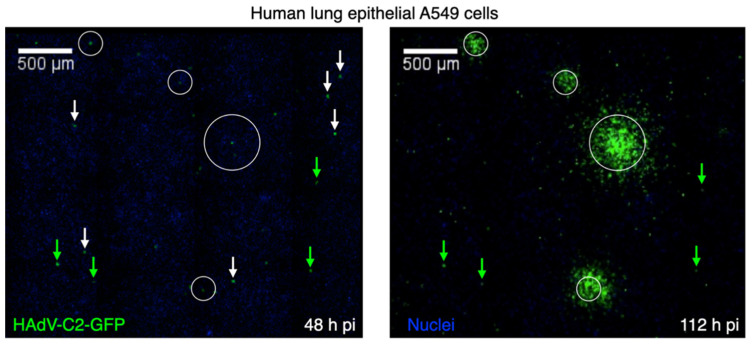
Cell-to-cell variability in plaque formation by HAdV-C2 revealed by single-cell time lapse fluorescence microscopy. Virus-infected cells and infection foci were visualized by EGFP expressed from the viral genome. Live cell plaque assay was conducted under agarose conditions, which restrict convectional spreading of cell-free extracellular virus particles and give rise to round plaque phenotypes, as described in [11]. Circled areas contain plaquing infections, green arrows indicate persistently infected non-plaquing cells, and white arrows indicate infected cells that were eliminated from the dish between 48 and 112 h pi. Nuclei are stained with Hoechst (weak signal in blue). Data from reference [11].

Recent advances in single-cell analyses, such as single-cell RNA sequencing (scRNA-seq), single-cell, single-molecule RNA fluorescence in situ hybridization (scRNA-FISH), high-throughput fluorescence in situ DNA hybridization, and single-cell mass cytometry, have unambiguously demonstrated that biology is highly heterogeneous at the single-cell level [7,8]. This heterogeneity not only applies to different cell types in a tissue but is also observed with clonal tissue culture cells (reviewed in [9,10]). Clearly, virus infections of single cells proceed with different kinetics and have different outcomes. Impressively, this has been visualized by a second-generation plaque assay, Plaque 2.0, in live cell mode using fluorescent viruses, such as human adenovirus (HAdV) (see Figure 2, and [11,12,13,14]).

In this review, we highlight emerging techniques in single-cell analyses of virus infections by focusing on a set of scRNA-seq and single-cell fluorescence microscopy studies in traditional tissue culture cancer cells, differentiated tissue models, and mice and humans. We use seven different viruses, namely HAdV, coxsackie virus (CV), influenza A virus (IAV), dengue virus (DENV), zika virus (ZIKV), herpes simplex virus (HSV), and sendai virus (SeV), as examples to show how single-cell studies have provided new insights into virus–host interactions. For aspects of single-cell investigations in other viruses, we refer the reader elsewhere—human immunodeficiency virus infections [15,16], human cytomegalovirus latency [17], and vaccine development, diagnostics, or therapeutics [18,19]. For coronavirus single-cell infection analyses, the reader may consider recent reviews [20,21,22,23].

## 2. Analyzing Single Infected Cells

Single-cell infection studies by scRNA-seq have recently become prominent and have revolutionized gene expression studies in virus infections. The power of scRNA-seq lies in the delivery of simultaneous snapshots of both virus and host transcriptomes. However, scRNA-seq studies are not simple to perform and analyze, and require isolation of single cells, RNA amplification, and complex data analysis. Information on the different scRNA-seq procedures, as well as technical and computational challenges in their implementation, has been provided elsewhere [24,25,26,27].

Single-cell, single-molecule RNA-FISH combined with fluorescence microscopy is an alternative to scRNA-seq to analyze gene expression. This procedure identifies mRNAs via specific oligonucleotide probes. Either multiple fluorophore-conjugated probes or multiple probes combined with a signal amplification system, such as branched DNA technology, allow visualization of individual mRNAs as fluorescent puncta (for example, [28]). Single-molecule RNA-FISH has a high degree of sensitivity and extends to single mRNAs at nanometer localization precision. However, due to the limited number of fluorophores simultaneously applicable in fluorescence microscopy, traditional single-molecule RNA-FISH monitors only about four different transcripts at a time, although some multiplexing can be achieved by sequential rounds of hybridization [29,30]. Single-molecule RNA-FISH protocols can be combined with either immunofluorescence staining for proteins or with copper(I)-catalyzed azide-alkyne cycloaddition (click) reaction for staining of ethynyl-deoxy-nucleotide-tagged DNA [31]. Together with single virion tracking in live and fixed cells, this has laid the foundation for analyzing the variability of HAdV entry into cells, including virions, as well as viral DNA and transcripts [32,33,34,35,36].

**Figure 3:**
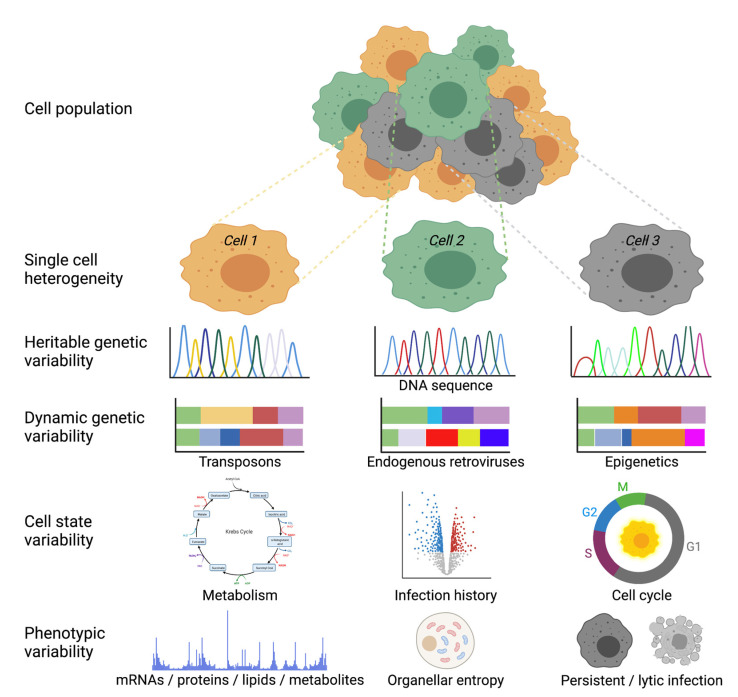
Cell-centric mechanisms underlying virus infection variability. Cells in a given population may be variable at the single-cell level due to at least four principle features: one is heritable genetic variability in the DNA sequence; two is dynamic genetic changes driven by mobile genetic elements or epigenetic differences; three is cell state differences, such as metabolism, infection history, or cell cycle; four is differences in phenotypes, such as macromolecules, organellar positioning with respect to each other (entropy), or the infection state. Figure was prepared by using BioRender.

A distinct limitation of both scRNA-seq and RNA-FISH technologies is that they provide snapshot views into infection; although, recently, single-cell thiol-(SH)-linked alkylation of RNA for metabolic labelling sequencing was introduced to differentiate between new and old RNA [37]. Live cell fluorescence microscopy, in contrast, allows for long term tracking of a particular infected cell, but with a limited number of molecular markers. Live cell fluorescence reporter systems are available to analyze both transcription and translation processes. They function through the recruitment of a fluorescent reporter protein to a specific target structure on an mRNA or a nascent protein [38,39,40]. Below, we discuss some of the insights derived from single-cell studies of particular viruses. The overall concepts, from virus-centric and cell-centric points of view, are presented in Figure 1 and Figure 3, respectively.

## 3. Single-Cell Variability in Adenovirus Entry, Transcription, and Spreading

### 3.1. Entry

For decades, conventional plaque assay, immunofluorescence microscopy, and flow cytometry have all been useful for virus infection analyses at single-cell resolution [1,32,33,41]. They allow the tracking of single infected cells, as well as single virions and their genomes, and give insights into the mechanisms of entry and infection, as exemplified by HAdV, a nonenveloped double-stranded DNA virus that replicates in the cell nucleus [5,42]. In short, quantitative fluorescence microscopy and image analyses have revealed 10–15-fold cell-to-cell differences in virion binding to human epithelial cells or murine alveolar-like macrophages, yet only 2- to 3-fold differences in virion endocytosis and endosomal penetration, despite a 10-fold variability in the exposure of the internal membrane lytic protein VI from the incoming particles (Table 1). Protein VI causes disruption of the limiting endosomal membrane and thus mediates penetration of the HAdV from endosomes to the cytoplasm. The quantifications/numbers suggest that, as long as the virions expose protein VI, they penetrate the endosomal membrane. If they fail to expose protein VI, they do not reach the cytosol and are subject to degradation [43,44,45,46,47,48].

Arguably, the most variable intracellular step in HAdV entry is the nuclear import of viral DNA, which varies up to 15-fold between cells if analyzed by quantitative click chemistry (Table 1). Much of this variability is due to the cytosolic misdelivery of viral DNA upon virion disassembly at the nuclear pore complex [36,49]. Although immunostaining against the viral DNA-associated protein VII, a DNA binding and condensing protein, has been used as a proxy for viral DNA nuclear import, it is unsuitable for detecting misdelivered viral DNA [50,51,52]. Accordingly, the cell-to-cell variability in protein VII-based nuclear import is biased towards the detection of nuclear viral DNA with less cell-to-cell variability than when single viral genomes are analyzed by click chemistry (Table 1).

**Table 1:** Single entity assays reveal cell-to-cell variability in HAdV-C infection.

Infection Step	Variability	Evidence	References
Virus binding	10–15×	Fluorescent HAdV-C2/C5 virions; epithelial cells (CAR)/alveolar macrophages (SR-A6).	[53,54,55]
Endocytosis	2–3×	Fluorescent HAdV-C5 and immunofluorescence microscopy.	[44,56,57]
Protein VI exposure	10×	Intensity of immuno-stained protein VI on endocytosed virions at 10 or 20 min pi.	[44,58]
Penetration into the cytosol	2–3×	Streptolysin-O mediated plasma membrane permeabilization and staining of cytosolic HAdV-Alexa488 species B or C by perfused anti-Alexa488 antibody. Penetration inactive HAdV-C2_TS1-Alexa488 served as negative control for variability. Data are in agreement wth thin section electron microscopy resolving single virions in endosomes.	[44,47,48,59,60]
Nuclear targeting	low	Fluorescent HAdV-C2/C5 virions.	[49,61,62]
Uncoating of virion DNA	low	Fluorescent HAdV-C2/C5 virions and clickable virion DNA.	[49,63]
Nuclear import	3×/15×	Confocal microscopy of DNA-associated protein VII & clickable viral DNA. Note: the protein VII-based immuno-staining does not detect mis-delivered viral DNA in the cytosol, unlike click-staining.	[31,44,49]
E1A, E1B-55K early transcription	10–15×	scRNA-FISH, in combination with localization of incoming viral DNA by click chemistry.	[31]
Major late transcription	10–15×	scRNA-FISH in human lung epithelial cells.	[31]
DNA replication	Variable onset	Click chemistry and sc DNA-FISH.	[31]
Assembly	?	Co-assembly model of virions from components suggests that there is a large excess of unassembled over virion-incorporated capsomers.	[64,65]
Proteolytic maturation	10% light, 90% heavy particles	HAdV-C5 particles isolated from producer cells by CsCl density gradient centrifugation assays. Two bands are typically visible on the gradient: “light” particles with unprocessed structural proteins and infectious “heavy” particles with proteolytically processed structural proteins.	[66,67,68]
Egress	73% lytic; 27% nonlytic	Single well, single plaque assays by live cell imaging.	[42,69]
Lysis/Persistence		Simultaneous single-cell in situ analyses of HAdV-C5 gene expression, suppression of the E1A promoter by IFN, and activation by Ire1α/XBP1 axis of the unfolded protein response pathway.	[70,71,72]

### 3.2. Gene Expression

Several additional highly variable steps are downstream of entry. The first one is a 10- to 15-fold variability in early adenoviral gene expression. This is based on the observation that not all of the incoming genomes in the cell nucleus are transcriptionally active at any given time point [31]. This discovery was achieved by scRNA-FISH using an intronic probe of the early region 4 of the viral genome, which was large enough to yield a stable and robust signal colocalizing with the click-labeled viral genome in the nucleus [31]. This result was distinct from the E1A mRNA signal, which did not localize to the viral DNA, and was dispersed across the cytoplasm. There was no correlation between the number of viral genomes in the nucleus and the fraction of transcriptionally active viral DNA, indicating that abundant viral DNA in the nucleus does not sequester away factor(s) necessary for viral transcription. These data were mirrored by the observation that certain cells with 10 to 20 viral genomes expressed only a handful of E1A transcripts, while other cells with just a few genomes had hundreds of E1A transcripts. This was in agreement with the 10- to 15-fold differences in late viral gene expression, and a variable onset of viral DNA replication. How these large variations in viral gene expression relate to the lytic and persistent infection phenotypes observed by simultaneous single-cell in situ analyses of HAdV-C5 gene expression can now be analyzed [70]. Intriguing yet unresolved questions also comprise the cell variability in AdV progeny production and the extent of proteolytic processing of light immature to infectious mature particles (Table 1). Population data show that infected cultured cells produce a large excess of unassembled capsomers and, for the most part, give rise to heavy mature infectious particles and only about 10% light immature particles [66,67,68].

### 3.3. Virus Egress and Spreading

In addition to variability in viral entry, replication and assembly, there is a surprising duality in AdV egress phenotypes, as recently revealed by single plaque per well assays in live imaging mode [11,42]. This fluorescence imaging assay is conducted with a fully replicating GFP expressing HAdV-C2 in the absence of an agarose overlay on the indicator cell layer [11]. It is an extension of the classical plaque assay, with agarose yielding round plaques [1,73]. Unlike classical plaque assay, the “Plaque 2.0” imaging assay gives rise to both round and comet-shaped plaques [12]. The infected “founder” cells in the round plaques remain viable upon infection spread to the neighboring cells, while the founder cells of the comet-shaped plaques lyse. The round plaques were not inhibited by the presence of neutralizing antibodies in the culture medium, which suggests that they arise by a nonlytic transfer of progeny virus without cell-free virions. In accordance, the round plaques are not affected by convection forces in the medium, while comet-shaped plaques are driven by lytic virus release and convection flux in the medium [11]. Limiting dilutions of the purified HAdV-C2 inoculum gave rise to single plaque per well, with both small concentric and large comet-like shapes at a ratio of about 1:3 (Table 1, [42]). Interestingly, when viruses collected from the small plaques were inoculated to naïve cells, they readily formed both small, round plaques and comet-shaped ones, as in the original inoculum. This indicates that the small nonlytic plaques form independently of the large lytic plaques. This is likely due to cell intrinsic variability rather than genetic changes in the progeny. The recent development of machine-learning algorithms allows us to extract nuclear features from fluorescence microscopy and interrogate the molecular mechanisms of cell-to-cell variability in virus egress [69]. Intriguingly, convolutional neural networks revealed surprising differences between nonlytic and lytic infected cells. The latter accumulate structural viral proteins faster in the cell nucleus than the former, their nuclei are under higher relative pressure and their nuclear envelope is more fragile, as indicated by laser ablation and nuclear efflux measurements. This suggests that the envelope of lytic nuclei has both a lower mechanical strength and a higher pressure than the nonlytic one, a feature that can promote efficient nuclear lysis. The live cell imaging of HAdV-C2 infection also clearly shows that the lytic virus egress is a two-step process: first, the nuclear envelope ruptures releasing progeny into the cytoplasm, followed by progeny release into the culture medium 1–2 h later. This time difference between nuclear and plasma membrane rupture implies that the two events are driven by distinct signals and molecular machineries.

In addition to the cell biological differences between lytic and nonlytic infected cells, HAdV leads to persistent infections of lymphoid cells and fibroblasts; for example, in the presence of interferon (IFN) [70,71,72,74,75]. Persistence is maintained by the expression of the viral E3-19K glycoprotein, which activates the Ire1 unfolded protein response (UPR) sensor in the endoplasmic reticulum, and increases the levels of active transcription factor XBP1s, which maintains the expression of the viral immediate early gene E1A (Table 1). It will be interesting to explore the variability in E1A expression and correlate it to the activity of the UPR sensors and effectors, as well as the IFN signaling status of individual cells. Together, these findings testify that HAdV is part of a growing number of non-enveloped viruses that not only persist in infected cells but also spread by a dual egress mode, both lytic and nonlytic. The latter feature is especially well known in RNA viruses owing to their genetic and genomic instability [76,77].

## 4. How Early Events Give Rise to Cell-to-Cell Variability in Coxsackie Virus Infection

A recent study on early events of CVB3 (genus *Enterovirus*) infection identified the switch between translation and replication of incoming virus genome as one source of cell-to-cell variability in CV infection [78]. The CV genome is a single-stranded RNA molecule of positive polarity. The incoming genome is delivered into the cytoplasm and functions there as an mRNA to produce the viral replication machinery components, which then amplify the genome by a negative-strand copy. Boersma et al. [78] engineered the CVB3 genome to contain an array of SunTag peptide-coding sequences in-frame and upstream of the viral coding region. Expression of this genome enabled live cell imaging of genomic RNA translation in cells expressing an intracellular single-chain variable fragment fused to GFP and directed against the SunTag peptide. Viral RNAs in translation appeared as bright GFP fluorescence puncta because multiple ribosomes typically engage simultaneously with a single mRNA. The number of GFP puncta per cell increased over time, which was indicative of viral RNA replication.

### 4.1. Five Phases of CV Replication

Further analyses distinguished five phases of virus replication. During phase one, the incoming viral genomes are translated and a limited number of GFP dots is observed, typically only one at multiplicity of infection (MOI) 0.25. Phase two is marked by disappearance of the GFP signal, so this phase most likely represents a switch from translation to initial replication of the incoming genomes. Phase three starts with reappearance of GFP puncta, which rapidly increase in number, thus reflecting translation of newly replicated positive-strand genomes. During phase four, the number of GFP puncta remains rather constant, about 15–20 translating viral genomes per cell. This phase most likely prepares for a new round of replication since, in the following phase five, a second rapid increase in number of GFP puncta per cell is observed. The use of replication inhibitors and smFISH confirmed the increase in intracellular viral genomes from phase three onwards. Unfortunately, the efficiency of genome translation remains unexplored, although snapshots suggest that the majority of genomes also translate. It appears, however, that the fraction of actively translating vRNAs would differ between cells.

### 4.2. IFN Intercepts the Switch to Replication

Interestingly, although these five different phases were observed in most infected cells and in different cell types, the initial translation phase of the incoming viral RNAs varied widely between individual cells, from 12 min to more than 4 h [78]. As a result, the infected cells were readily observed in different phases of infection, even though the infection was synchronized by applying limited amounts of inoculum for a short time. Interestingly, 15–20% of infected cells at low MOI failed to switch to the replication mode, despite successful translation of the incoming genomes. The switch to the replication phase also turned out to be a major susceptibility point for IFN antiviral defense, since pretreatment of cells with IFNα2 significantly reduced the fraction of infected cells containing replicating viral genomes. Several IFN-stimulated genes (ISGs), and probably multiple mechanisms, account for this observation, as suggested by siRNA-mediated gene knockdown experiments. Overall, this study is a yet another testimony to the power of live cell infection studies.

## 5. Viral and Cellular Heterogeneity in Influenza a Virus Infection

RNA viruses have high genetic variability, which raises the question of the contribution of virus vs. host cell heterogeneity to infection variability. scRNA-seq experiments of influenza A virus (IAV) infection in standard tissue culture cells have explored this topic. The genome of influenza viruses consists of eight negative-sense viral RNA (vRNA) segments, with each segment carrying multiple copies of viral nucleoprotein (NP) and a single copy of viral polymerase complex composed of three proteins, PB2, PB1, and PA (reviewed in [79]). The complex of vRNA-NP-polymerase is referred to as the viral ribonucleoprotein (vRNP) complex. After fusion of the incoming virion envelope with endosomal membrane, the vRNPs are released into the cytoplasm and imported into the nucleus, which is the site of virus gene transcription and replication [80]. Each genome segment functions as a distinct transcription and replication unit. Primary transcription from the incoming vRNPs produces new vRNP protein components which, after translation in the cytoplasm, are imported back to the nucleus. These newly synthesized vRNP protein components, together with select host factors, drive replication of the vRNPs via a full-length complementary genomic copy (cRNA). Secondary transcription from the replicated genome segments yields the bulk of viral mRNAs in the infected cells.

Analyses of single MDCK cells indicated that progeny virus output and intracellular vRNA levels by RT-qPCR quantifications were highly variable between individual infected cells by up to three orders of magnitude [4]. Furthermore, protein expression analyses on single MDCK cells indicated that majority of IAV-infected cells failed to express a full set of viral proteins at low MOI [81]. Subsequent scRNA-seq studies have also confirmed the high cell-to-cell infection variability in human lung carcinoma A549 cells and primary bronchial epithelial cells [82,83,84,85,86].

### 5.1. Viral Factors

One reason for the high cell-to-cell variability in the production of infectious progeny, as well as viral transcripts per cell, could be the presence of defective interfering (DI) particles in the inoculum [86,87]. DI particles are common in RNA virus preparations passaged at high MOI. The genomes of DI particles carry internal deletions of variable length and/or point mutations that render them helper virus-dependent for replication but, at the same time, DI genomes interfere with full length virus genome replication and packaging since the aberrant DI genomes still retain packaging signals, as well as promoter and replication sequences [88]. Since IAV DI deletions frequently occur in polymerase subunit segments [86,89,90], DI genomes in low MOI infections are replication incompetent and cannot proceed to secondary transcription, and thus score as cells with low virus transcript numbers in comparison to cells with active viral genome replication. However, extreme variations in IAV transcript numbers per cell have also been reported in studies that (1) carefully limited the amount of DI particles by using virus inocula produced by low MOI, and (2) excluded trivial reasons for infection variability by using low MOI synchronized infections and restricted analyses to single round infections [84,85].

Apart from these technical points, the fraction of viral mRNAs compared to total mRNA in individual cells was anything from < 0.1% to over 50% at 8 h pi [84], or from <1% to about 90% at 16 h pi [85]. Furthermore, Russell et al. [84] found that less than 10% of infected cells contained over half of the scored viral transcripts at 8 h pi, thus underlying the importance of single-cell analyses over bulk assays. Although a significant fraction of infected cells in both studies lacked one or more viral transcripts, a wide range of virus transcript numbers was also observed in cells expressing all viral genes.

Influenza genomes are known for their high genetic diversity (reviewed in [91]). To assess the impact of IAV genetic variability on cell-to-cell infection heterogeneity, transcriptome analyses and full sequences of all virus genes from single A549 cells were employed, with a low diversity infection stock generated from plasmid DNA, limited virus passaging, and a moderate MOI infection, where about 25% of cells had viral transcripts [83]. Although cells expressing unmutated copies of all viral genome segments displayed overall less heterogeneity in virus load than cells with incomplete or mutated viral genes, both unmutated genomes and mutated genomes were able to drive high levels of gene expression, with virus transcripts ≥50% of cell transcriptome. Conversely, a full set of unmutated genes did not guarantee efficient expression of viral transcripts. Only the failure to express an RNP component clearly decreased the intracellular number of viral transcripts. Thus, genetic variation in virus populations only partially accounts for the heterogeneity of IAV gene expression at the single-cell level. It is unknown whether stochasticity or undiscovered host cell factors contribute to variability, such as endogenous retroviruses that the host may coopt to stimulate innate immunity against IAV, or, alternatively, IAV “unwittingly” creates a change in the host that unleashes endogenous retroviruses to enhance a host’s innate immune responses to IAV [92].

### 5.2. Cellular Factors

Another source of variability could be IAV entry into cells. For example, differences in the nuclear import efficiency of incoming vRNP segments could be one reason for the failure to express all virus genome segments in a cell. The nuclear import of incoming vRNPs has been studied by single-molecule FISH (smFISH) with probes targeting two different segments and live imaging of viruses with dual-color quantum dot-conjugated vRNPs [93,94]. Interestingly, the two studies do not agree on whether the incoming genome segments travel as one bundle to the nuclear envelope or whether the segments are separate already in the cytoplasm following fusion of the virion envelope with the endosomal membrane. Possibly, this discrepancy is owed to variability in abundance of the vRNP debundling factor transportin 1, which removes matrix protein 1 from the RNPs [95]. Debundling prior to delivery to the nuclear envelope could lead to asynchronous import of the RNP genomes into the nucleus [94]. This could, in turn, contribute to variation in the packaging accuracy during virion assembly, although the accuracy is also strain-dependent and affected by mutations in the nucleocapsid protein [96,97]. In addition, the incoming IAV vRNPs disperse randomly in the nucleus, and are likely in different nuclear microenvironments [93,94]. Collectively, different cellular microenvironments of vRNPs impact viral transcription, replication, and packaging efficiencies.

## 6. Host Transcriptome Changes in Virus Infections: DENV, ZIKV, and HSV-1 as Examples

### 6.1. DENV and ZIKV Infections

Infection of tissue culture cells by single-stranded positive-sense RNA viruses can result in large differences in viral RNA copy numbers, as demonstrated by scRNA-seq analyses of *Flaviviruses* West Nile virus (WNV)-infected murine fibroblast L929 [98], DENV-, or ZIKV-infected human hapatoma Huh7 cells [99]. scRNA-seq data allow comparison of host transcriptomes between cells with low and high viral loads. Differences could point towards potential pro-viral host gene expression patterns or molecular pathways. However, caution is required, since scRNA-seq, even when several time points post infection (pi) are considered, does not allow time-resolved analysis of the same cell. It is clear from the non-infected controls that heterogeneity in host transcriptome exists prior to infection. How this preexisting heterogeneity affects the infection process at a given time point pi cannot be read from scRNA-seq measurements. Thus, scRNA-seq data do not tell what host features in the early infection cycle lead to efficient virus replication later on. Validation experiments are needed to strengthen the scRNA-seq correlations.

Zanini et al. performed validation experiments for multi-time point single-cell RNA-seq profiling of human hepatoma (Huh7) cells infected with DENV or ZIKV flaviviruses at 4, 12, 24, and 48 h pi [99]. They determined Spearman’s rank correlation coefficients for expression of each gene and intracellular virus transcript load, and discovered host genes that showed increased expression in cells with high intracellular virus abundance in both viruses (e.g., genes of the PERK branch of the UPR), but also genes that showed opposing expression trends for the two viruses; for example, the molecular chaperone HSPA5. Furthermore, some host genes displayed opposing expression trends, with intracellular virus loads at early compared to later time points. Loss-of-function by siRNA and gain-of-function experiments probed the functional relevance of select genes that correlated or anticorrelated with high DENV viral loads, and revealed previously unknown DENV pro-viral genes, such as RPL31, TRAM1, and TMED2, and antiviral ones, including ID2 and CTNNB1 [99,100]. The question arises as to whether extreme changes in gene expression induced by genome-wide siRNA or CRISPR-Cas9 knock-out screens correlate to “physiological” expression variability of pro- or antiviral factors, which would equally affect intracellular viral loads at single-cell levels. Answers to this question are still largely buried in scRNA-seq data. A study with DENV and ZIKV infections demonstrated that a few of the host genes picked from scRNA-seq data were previously identified as essential flavivirus host factors in CRISPR-Cas9 screens [99]. Another study with IAV, however, noted that none of the host genes significantly associated with high viral transcript loads were previously identified in genome-wide screens of IAV replication [84].

### 6.2. HSV-1 Infection

A recent study of single-cell transcriptional heterogeneity discovered a host signaling pathway linked to efficient HSV-1 infection [101]. HSV-1 is a double-stranded DNA virus that replicates in the host cell nucleus. scRNA-seq analysis of HSV-1-infected primary human fibroblasts at 5 h pi (MOI 2, about 70 genomes per cell) identified significantly upregulated host genes in highly infected single cells, comprising genes in reprogramming towards an embryonic-like transcriptional state, including the WNT/β-catenin pathway. Reprogramming involved the recruitment of β-catenin to the host nucleus and viral replication compartments, and was required for late viral gene expression and progeny production. Noticeably, the WNT/β-catenin pathway plays a central role in development, tissue maintenance, and cancer development [102,103]. Abortively infected cells, on the contrary, exhibited strong antiviral signalling, a feature absent in the highly infected cells.

## 7. Cell-to-Cell Variable Interferon Response in IAV and SeV Infections

Virus infection induces dynamic changes in the host transcriptome, affecting both the response against the infection and the virus-tinkered response. A central response of the host against virus is the induction of the IFN response. Induction starts by recognition of pathogen-associated molecular patterns (mostly nucleic acids) by host pattern recognition receptors, such as the endosomal Toll-like receptors [104], cytosolic RNA-sensing retinoic acid-inducible gene I (RIG-I)-like receptors [105], or the cytosolic DNA-sensor cyclic GMP-AMP synthase (cGAS) [106]. Pattern recognition receptors trigger signaling cascades and induce transcription factors that upregulate the expression and secretion of IFNs and other cytokines. IFNs, in turn, induce expression of multiple ISGs, both in the infected cells (autocrine signaling) and in non-infected cells (paracrine signaling), leading to restriction of infection spread in the host [107]. To evade this host defense, viruses suppress host gene expression and/or encode proteins that counteract different steps of the IFN response cascade [108].

### 7.1. Influenza Virus

The main pathogen sensor for influenza is RIG-I, especially for defective genomes and possibly cellular dsRNAs that are upregulated by the infection [92,109,110]. The main viral antagonist of IFN induction is NS1, and additional virus proteins contribute to dampening of the IFN response, such as the viral polymerase complex proteins, as well as PA-X and PB1-F2, products of alternative reading frames of the segments 3 and 2, respectively. Recent scRNA-seq studies have revealed that the intracellular virus load is the key feature correlating with upregulation of IFN transcripts at a single-cell level. Three studies in A549 cells reported that high levels of IFN induction correlated with the failure to express NS-segments in infected cells [82,83,85]. They all used single round infections at time points between 12 to 16 h pi. The correlation was, however, strain-specific with the seasonal H3N2 IAV, but not with the 2009 pandemic H1N1 strain [85]. Due to the biphasic nature of IFNβ induction, where the first early responder cells secrete IFN and upregulate ISGs, such as RIG-I, infection level and ISG expression do not necessarily correlate due to bystander effects, especially at early time points.

Population analyses of A549 cells suggest that an increase in bulk MOI leads to more efficient type III (IFNL1), but not type I (IFNB1), induction, especially at early time points of IAV infection [111]. One possible explanation is that increasing MOI leads to more rapid virus replication and accumulation of RIG-Iigands. However, single-cell analyses reported that viral transcript counts did not differ significantly between IFN^+^ and IFN^-^ cells that expressed the NS segment, but a high virus load was more likely to activate IFN genes than a low virus load in the cells lacking NS expression [83]. This argues that increasing levels of pathogen patterns increase IFN production, unless blunted by NS. However, NS deficiency was not deterministic for IFN-I and IFN-III induction by viruses lacking large parts of PB2, which strongly induced IFN-I and IFN-III, as shown in scRNA-seq [86]. Thus, although specific genetic defects appear to increase the probability of IFN induction in influenza infection, variation in virus population only partially explains the heterogeneity of induction at a single-cell level.

### 7.2. SeV Infections

One intriguing feature of the host IFN response is that only a small fraction of infected cells actually upregulates IFN genes (reviewed in [112]). Factors postulated to contribute to this heterogeneity are variable intracellular levels of the pattern recognition receptors and signaling pathway components, leading to upregulation of IFN genes, variable virus loads, and virus genetic variability [113,114].

However, how well do these features correlate with the IFN induction at single-cell levels? Analyses of early IFNB1 gene induction by single-molecule RNA-FISH was conducted in HepG2 cells infected with DI-rich SeV stocks [115]. DI-genomes of the single-stranded RNA virus SeV are especially good ligands for RIG-I [116]. The study found that SeV amplified the expression of a subset of ISGs, including RIG-I and MDA5, in a fraction of infected cells at an early time point, prior to the appearance of IFNB1 transcripts; this is in line with previous observations that a subset of ISGs can be induced in an IFN-independent manner [117,118]. When IFN expression became detectable, a good correlation between the number of RIG-I and IFNβ transcripts at single-cell level was observed (Pearson’s correlation coefficient 0.74). Yet, viral L gene transcripts were largely uncorrelated with the IFNβ transcript numbers (Pearson’s correlation coefficient 0.26). Furthermore, the results suggested that early amplification of RIG-I or other ISGs in a limited number of virus-infected cells could initially activate IFN responder cells, since the abundance of virus sensors and transcription factors of IFNβ induction positively correlated with IFNβ induction [114]. However, we notice that not all SeV-infected cells that underwent early amplification of RIG-I transcripts turned into IFNβ-expressing cells.

## 8. Emerging Question One—Which Cells Are Virus-Targeted in a Tissue?

A range of single-cell analyses in tissue culture cancer cells have shown that both viral and cellular factors affect the cell-to-cell heterogeneity of virus infection. The picture becomes even more complicated when the focus is shifted from single-cell types to tissue infections. Tissue consists of different cell types with complex inter-cell communication. scRNA-seq studies with influenza have been conducted in differentiated pseudostratified airway epithelia grown at air–liquid interface [119], in mouse models [120,121], or human samples [122]. These studies detected virus RNA at significant levels not only in all subtypes of epithelial cells but also in neutrophiles, macrophages, fibroblasts, and T lymphocytes. The highest intracellular viral loads were in epithelial cells, with notably large cell-to-cell variability. The pseudostratified airway epithelium in large airways contains four main cell types: basal, secretory, goblet, and ciliated cells. Initial immunofluorescence studies in differentiated air–liquid interface tracheal/bronchial cell cultures showed that nonciliated cells are the primary targets for human IAVs, whereas avian IAVs only infect ciliated cells [123,124]. This difference correlated with the distribution of virus receptors, the alpha2–6-linked sialic acid for human viruses, and the alpha2–3-linked sialic acid for avian viruses. The scRNA-seq studies have provided a deeper view into the infected cell types, showing that secretory and ciliated cells have the highest viral loads in progressed human IAV infections [119,122].

## 9. Emerging Question Two—Which Cells Are the Main Interferon Producers in IAV Infection?

A comparison of IFN transcripts in the nasal wash cells of influenza-infected patients indicated that infected epithelial cells were the main producers of both type I and type III IFN mRNAs [122]. In the pseudostratified air–liquid culture of tracheal/bronchial cells, the pandemic IAV H1N1pdm09 predominantly upregulated IFN-III transcripts [119]. However, no distinct epithelial cell type stood out as an IFN producer, and no significant correlation between IFN expression and intracellular virus load was observed, despite an overall robust ISG induction in both infected and bystander cells, albeit with cell type-dependent differences in particular ISGs. Variable induction of ISGs in different cell types has also been observed in influenza-infected murine lung cells [125]. ISGs restrict virus replication and provide an important shield for uninfected bystander cells [107].

However, a recent study suggests that efficiency of this shield is not the same within different cell types of the respiratory track. When mice were sequentially inoculated with two IAV reporter viruses encoding either GFP or mCherry, or with each virus alone, the frequency of infected type I alveolar cells, which are involved in gas exchange between the alveolar lumen and the blood, was similar between single and sequential infections, but a clear reduction in infection efficiency for the second virus in sequential infections was observed in ciliated epithelial cells [126]. Similar results were obtained with mice treated with IFNβ or IFNλ prior to single virus infections. The reason why type I alveolar cells remain susceptible to IAV infection is unknown, especially since these cells upregulate ISGs upon IAV infection [127]. We speculate that the type 1 alveolar cells lack the distinct intrinsic defense normally present in polarized epithelial cells or professional antigen-presenting cells, such as endosomal acid ceramidase converting ceramide into sphingosine and thereby increasing intralumenal vesicles, which trap incoming enveloped viruses [128]. The lack of intrinsic defense would make these cells vulnerable to infection so that innate immunity would not sufficiently protect them.

## 10. Conclusions and Outlook

Single-cell studies provide valuable new insights into virus life cycles. They contribute to resolving the three major outcomes of virus–host interactions, i.e., productive infection yielding high amounts of progeny virions, persistent infection with low amounts of progeny, and abortive infections lacking progeny formation (see Figure 3). Single-cell studies at the level of organoids and tissues address the question how virus infections are tuned by accessory factors of the immune system, including specialized immune cells and their chemokines and cytokines, including IFN. Apart from IFN, secreted cytokines and chemokines establish the communication between a range of different cell types and regulate the entry of immune cells into an infected tissue, such as the lung. Innate immune responses, together with cell intrinsic factors, restrict virus spread and activate adaptive immune responses, which eventually lead to clearance of the virus. A tightly balanced immune response is crucial, and an exaggerated response can lead to severe tissue damage [129,130].

This review has shown that there are no simple explanations for infection variability at single-cell levels. Virus and cellular factors together, such as in IAV infection, or predominantly cellular factors, as discussed for HAdV infection, contribute to cell-to-cell infection variability. As exemplified by live imaging of CVB3, distinct steps in the virus life cycle can have highly variable durations, and this variability is most likely an important factor in determining cell-to-cell differences at the level of virus transcript abundance. Thus far, single-cell studies have exposed only a fraction of the cell-to-cell variabilities in virus infections. New technologies are emerging, such as a simultaneous high-throughput method for the measurement of chromatin accessibility and mRNA counts (SHARE-seq) in single cells [131], expansion sequencing (ExSeq) for spatially accurate in situ RNA sequencing of thousands of genes in intact tissue [132,133,134], and improved in vitro lung models for respiratory viruses [135,136]. Progress is also being made in multi-omics analyses of single cells [137], as well as single-cell metabolomics profiling [138].

Expanding the single-cell omics view of virus infections is important for refining the picture of cell-to-cell variability in infected cells, as transcript counts do not necessarily predict protein levels or protein functional diversity arising from post-translational modifications [139]. This is especially important for viruses, which rewire host translation and modulate posttranslational modifications [140,141]. However, omics studies alone will not provide a mechanistic understanding of variability. Other procedures with single-particle, single-genome resolution, preferably in live cell mode, are required to reveal cause–effect relationships at the molecular level, and give insight into the mechanisms driving virus propagation, restriction into persistence, or elimination.

We envision that cell-to-cell variability of virus infection will be increasingly interrogated by computational modelling. For example, the classical Luria–Delbrück fluctuation test deciphers reversible switching between cell states and describes phenotypic switching of cancer cells in a population of drug-sensitive and -tolerant cells [142,143]. The model has recently been used to describe HIV reactivation upon tissue necrosis factor alpha treatment of cells containing single HIV integration events [144]. The contention is that, if cellular responses are purely random, the fraction of reactivating cells should have minimal colony-to-colony fluctuations given the large number of cells present after weeks of colony growth. In contrast, data show considerable colony-to-colony fluctuations, with the fraction of reactivating cells in a skewed distribution arguing for a non-random fluctuation. Such analyses will uncover the existence of a heritable memory cell state that regulates virus reactivation and contributes to infection variability (Figure 3). We postulate that single cells are in a state responsive to infection for a certain time, and that they then switch back to an irresponsive state that resists infection. Extending this notion to a clonal cell population, one can argue that pre-existing states in single cells are not entirely based on genetic factors but also on non-genetic factors; this is akin to phenotypes in rare diseases, as well as cancer.

## Data Availability

Not applicable.
